# Papillary Fibroelastoma Arising From the Coumadin Ridge: A Rare Extravalvular Cardiac Tumor Diagnosed by Multimodality Imaging

**DOI:** 10.7759/cureus.111107

**Published:** 2026-06-18

**Authors:** Dharmikkumar Jadvani, Aakash Rana, Rutvikkumar Jadvani, Jack Xu

**Affiliations:** 1 Internal Medicine, University of Arkansas for Medical Sciences, Little Rock, USA; 2 Medicine, Central Arkansas Veterans Healthcare System, Little Rock, USA; 3 Internal Medicine, Government Medical College, Surat, IND; 4 Cardiology, Novant Health, Winston-Salem, USA

**Keywords:** cardiac mass tumor, coumadin ridge, methicillin-resistant staphylococcus aureus (mrsa), multimodality cardiac imaging, papillary fibroelastoma, transesophageal echocardiogram

## Abstract

Papillary fibroelastoma (PFE) is the most common benign primary cardiac tumor in adults, yet its occurrence on the Coumadin ridge remains exceptionally rare. Here, we report a case of an 86-year-old woman with paroxysmal atrial fibrillation, sick sinus syndrome, and a dual-chamber pacemaker who was found to have methicillin-resistant *Staphylococcus aureus* (MRSA) bacteremia. On transesophageal echocardiography (TEE), an 11 × 9 mm highly mobile, pedunculated mass with frond-like appearance was found attached to the Coumadin ridge that had the characteristic of PFE. A prior cardiac CT from 2021 confirmed that the mass had been present for at least four years, ruling out acute infective endocarditis. Given the patient’s advanced age and significant comorbidities, surgical resection was deferred in favor of conservative management with serial imaging surveillance. This case highlights the importance of multimodality cardiac imaging (particularly three-dimensional TEE and cardiac CT) in characterizing cardiac masses, distinguishing PFE from endocarditic vegetations, and guiding individualized management in high-surgical-risk patients.

## Introduction

Primary cardiac tumors are uncommon, with autopsy series reporting a prevalence of 0.0017%-0.019%, most of which are benign [1]. Among these, papillary fibroelastoma (PFE) is the most common benign primary cardiac tumor in adults. Although PFEs predominantly arise from valvular surfaces (70%-88% of cases), they can occur on any endocardium-lined surface, with non-valvular locations accounting for 12%-30% of cases [2,3]. The Coumadin ridge represents an infrequent but recognized location for PFE. The Coumadin ridge (left lateral ridge or warfarin ridge) is a normal anatomic variant located in the left atrium between the left atrial appendage (LAA) and the left superior pulmonary vein. This band-like structure is formed by the coalition of the left superior pulmonary vein and LAA, resulting in a lateral fold of left atrial wall tissue that contains the ligament of Marshall, autonomic nerve bundles, and small atrial arteries [4].

The clinical presentation of PFE varies considerably. Many patients are asymptomatic, and lesions are often detected incidentally during echocardiography or cardiac imaging performed for other indications. When symptomatic, the most common manifestations are embolic events, followed by heart failure, and rarely sudden cardiac death [2,5]. Embolic episodes occur in 13%-32% of patients at presentation, with cerebral embolization being the most frequent, manifesting as stroke or transient ischemic attack (TIA) [2,5]. Left-sided PFEs can cause territorial infarction affecting the brain, retina, kidneys, mesentery, or limbs, while right-sided lesions may result in pulmonary embolism and subsequent pulmonary hypertension. The mechanism of embolization is thought to be associated with the tumor's friable papillary fronds, which can fragment or serve as a nidus for thrombus formation [6].

Valvular and non-valvular PFEs carry similar embolic complications, with tumor mobility, rather than size or location, identified as the only independent predictor of PFE-related death or non-fatal embolization [7]. One review of 725 PFE cases identified only 10 tumors arising from the left atrium, with just a few involving the ridge between the LAA and the left superior pulmonary vein [8]. Similarly, only a handful of case reports have been published describing Coumadin ridge PFE, highlighting the rarity of this anatomical location. Due to the rarity of PFE arising from the Coumadin ridge and its difficult location, accurate diagnosis with multimodality imaging is important. Here, we describe a case of PFE originating from the Coumadin ridge, detected incidentally during evaluation for bacteremia, illustrating its clinical significance, imaging findings, and clinical management.

After reviewing this case, readers will recognize PFE arising from the Coumadin ridge as an exceptionally rare extravalvular cardiac tumor, understand how multimodality imaging, including three-dimensional (3D) transesophageal echocardiography (TEE) and cardiac CT, differentiates it from infective endocarditis vegetations, and appreciate the role of individualized risk stratification in guiding conservative surveillance over surgery in high-risk patients.

## Case presentation

An 86-year-old woman with a past medical history of paroxysmal atrial fibrillation, sick sinus syndrome status post dual-chamber pacemaker implantation, non-sustained ventricular tachycardia, and sarcoidosis initially presented to an outside hospital with syncope due to orthostatic hypotension, and her amlodipine was stopped. During the workup, intermittent supraventricular tachycardia was noted on telemetry. Blood cultures obtained as part of the workup grew methicillin-resistant *Staphylococcus aureus* (MRSA). A transthoracic echocardiogram (TTE) showed preserved left ventricular systolic function without evidence of valvular vegetations. Chest radiography showed possible pneumonia, and urine cultures were also positive for *Staphylococcus* species. Given the presence of MRSA bacteremia and concern for a potential pacemaker lead infection, the patient was transferred to our institution for further evaluation. On arrival, she denied fever, chills, or recent illness and had no complaints related to her pacemaker pocket site, including erythema, warmth, or swelling. She was hemodynamically stable, well-appearing, and in no acute distress.

A TEE performed on this admission revealed a moderately sized, highly mobile echodense mass measuring approximately 11 × 9 mm attached to the Coumadin ridge with frond-like mobility and echocardiographic characteristics suggestive of a PFE (Videos 1, 2).

**Video 1:** Two-dimensional transesophageal echocardiogram (TEE) demonstrating a highly mobile, pedunculated mass with frond-like motion attached to the Coumadin ridge.

**Video 2:** Transesophageal echocardiogram (TEE) confirming the pedunculated, mobile mass arising from the Coumadin ridge.

3D live TEE imaging further confirmed the presence of a pedunculated mobile mass attached to the Coumadin ridge (Videos 3, 4).

**Video 3:** Three-dimensional transesophageal echocardiogram (TEE) characterizing the papillary fibroelastoma’s frond-like surface and pendulous motion during the cardiac cycle, illustrating the high degree of tumor mobility that confers embolic risk.

**Video 4:** Three-dimensional TruVue transesophageal echocardiogram (TEE) demonstrating the mass with enhanced tissue characterization. The TruVue rendering highlights the tumor’s anemone-like papillary fronds and confirms its origin from the Coumadin ridge, distinguishing it morphologically from a thrombus or vegetation.

A cardiac CT performed in 2021 was reviewed and showed the identical mass in the same location, confirming its chronic nature and excluding acute endocarditis (Figure 1).

**Figure 1:**
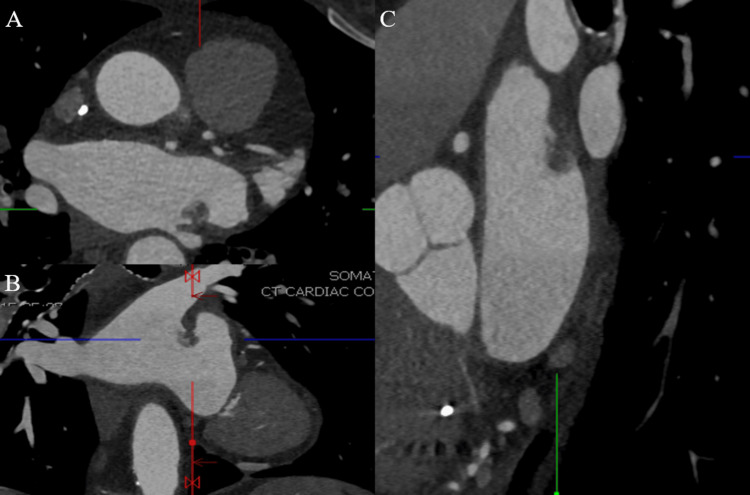
Cardiac CT multiplanar reconstruction demonstrating a small soft-tissue hypodense mass at the Coumadin ridge. The axial (A), coronal (B), and sagittal (C) planes confirm the lesion’s location and stable chronic appearance, consistent with a papillary fibroelastoma.

Infectious disease was consulted and recommended continuation of intravenous daptomycin for a six-week course of MRSA bacteremia once culture became negative. Cardiothoracic surgery was consulted; however, given the patient’s age and comorbidities, she was deemed a poor surgical candidate, and no operative intervention was pursued at this time. The patient was managed conservatively.

## Discussion

This case illustrates several important clinical principles. First, the presence of MRSA bacteremia required careful evaluation to rule out endocarditis. The availability of prior cardiac CT imaging confirmed the chronicity of the mass, which was invaluable in identifying the lesion as a pre-existing PFE rather than a vegetation. Second, the location on the Coumadin ridge, while uncommon, is a recognized site for PFE and requires multimodality imaging, including 3D TEE, for accurate characterization. Third, the patient's advanced age and comorbidities made surgical risk prohibitive, highlighting the need for individualized decision-making that balances embolic risk against operative risk. The highly mobile nature of this patient's PFE is concerning, given that mobility is the only independent predictor of embolic events [7]. However, the patient had no prior history of embolic phenomena despite the tumor being present for at least four years (documented on 2021 CT). This finding is supported by the data showing that approximately one-third of PFE patients remain asymptomatic [9]. Close clinical follow-up with serial echocardiography and heightened vigilance of embolic symptoms represents a reasonable approach for this high-risk surgical patient [9].

On echocardiography, PFEs typically appear as small, mobile masses with a homogeneous speckled pattern and characteristic stippling along the edges, often described as having a "frond-like" or "sea anemone" appearance. The sensitivity of TTE for PFE detection is approximately 62%, while TEE has 77% sensitivity [9]. 3D echocardiography, as used in this case, provides enhanced spatial resolution and can better delineate the tumor's attachment site and mobility. The presence of the identical mass on cardiac CT from 2021 in this case was crucial in excluding acute endocarditis and confirming the chronic, benign nature of the lesion. Recent studies suggest that nodule stalks on CT are pathognomonic for PFEs and help distinguish them from vegetations, though small lesions (≤7 mm) may pose diagnostic challenges [10].

The differential diagnosis of a mobile mass on the Coumadin ridge includes left atrial thrombus, cardiac myxomas, valvular vegetations in the setting of endocarditis, Lambl's excrescences (linear echocardiographic densities on valve edges), and metastatic tumors [9]. The prognosis following surgical excision is generally excellent, with low recurrence rates and minimal perioperative mortality [1]. However, management of non-valvular PFEs, particularly in elderly or high-risk patients, may vary depending on tumor mobility, embolic risk, and overall surgical candidacy [5,11].

A landmark Mayo Clinic study of 511 patients demonstrated that for those not undergoing surgical resection, the risk of cerebrovascular accident (CVA) was 6% at one year and 13% at five years [2]. In contrast, surgically treated patients had lower CVA rates of 2% at one year and 8% at five years [2]. The efficacy of anticoagulation or antiplatelet therapy for PFE remains uncertain. The Mayo Clinic study found no significant difference in subsequent stroke occurrence between patients treated with warfarin or antiplatelet drugs versus those not taking medication [2]. Among 121 patients with a history of neurologic events, 72% were on anticoagulant medications at PFE diagnosis (warfarin 22%, aspirin 47%), yet this did not appear to provide protection against recurrent events [2]. Despite the lack of randomized controlled trial data, some authors suggest that symptomatic patients who are not surgical candidates could be offered long-term oral anticoagulation, though its efficacy remains unproven [11]. One case report described successful conservative management of a left ventricular PFE with warfarin anticoagulation for over four years without complications [12].

## Conclusions

PFE arising from the Coumadin ridge is an exceedingly rare occurrence, which can be difficult to diagnose due to its unusual location and the need to rule out other, more common and life-threatening conditions, such as infective endocarditis. This case highlights several important clinical lessons. First, multimodality imaging (two-dimensional (2D) and 3D TTE and cardiac CT) should be used to accurately characterize and anatomically localize cardiac masses in this area. Second, comparison with prior cardiac imaging, when available, is crucial to help identify the lesion's chronicity and differentiate a benign neoplasm from an acute vegetation. Third, management of PFE should have an individual approach: while surgical resection is generally most effective to prevent future embolic events, conservative management is a reasonable strategy for elderly patients with high operative risk, particularly in patients with no prior embolization history. Clinicians should be aware of the Coumadin ridge as a potential site for PFE, as timely diagnosis and appropriate risk stratification are essential to improve patient outcomes.
